# Identification and Characterization of the Succinate Dehydrogenase Complex Iron Sulfur Subunit B Gene in the Oriental River Prawn, *Macrobrachium nipponense*

**DOI:** 10.3389/fgene.2021.698318

**Published:** 2021-07-15

**Authors:** Shubo Jin, Yuning Hu, Hongtuo Fu, Sufei Jiang, Yiwei Xiong, Hui Qiao, Wenyi Zhang, Yongsheng Gong, Yan Wu

**Affiliations:** ^1^Key Laboratory of Freshwater Fisheries and Germplasm Resources Utilization, Ministry of Agriculture, Freshwater Fisheries Research Center, Chinese Academy of Fishery Sciences, Wuxi, China; ^2^Wuxi Fisheries College, Nanjing Agricultural University, Wuxi, China

**Keywords:** *Macrobrachium nipponense*, *SDHB*, qPCR analysis, RNAi, male sexual development

## Abstract

Previous studies have revealed that *SDHB* has potential functions in the male sexual differentiation and development in *M*. *nipponense* through providing ATP. In this study, the functions of *Mn-SDHB* were further analyzed in depth using quantitative polymerase chain reaction (qPCR), *in situ* hybridization, western-blot, and RNA interference (RNAi), combined with the histological observations. The full-genome sequence of *Mn-SDHB* was 54,608 bp at Chromosome 34, including 7 introns and 6 exons. The full-length cDNA sequence of *Mn-SDHB* was 1,268 base pairs (bp) long with an open reading frame of 807 bp, encoding for 268 amino acids. The highest expression level of *Mn-SDHB* in different tissues was observed in the testis, and male prawns at post-larval developmental stage 25 during different developmental stages, indicating that *SDHB* was potentially involved in the male sexual development in *M*. *nipponense*. *In situ* hybridization and western-blot analysis indicated that *SDHB* plays essential roles in the testis development. The *in situ* hybridization analysis also implies the potential roles of *Mn-SDHB* in ovarian development. The expressions of *Mn-IAG* were decreased after *Mn-SDHB* dsRNA injection, indicating *SDHB* has the positive regulatory effects on *IAG* in *M*. *nipponese*. Thus, *SDHB* was involved in the mechanism of the male sexual development. The testis development was inhibited, and sperms were rarely observed after 10 days of *Mn-SDHB* dsRNA injection, indicating *SDHB* has positive effects on the male sexual development in *M*. *nipponense*. This study highlights the functions of *SDHB* in *M*. *nipponense*, which provide new insights for the future studies of the male sexual development in other crustacean species.

## Introduction

The oriental river prawn, *Macrobrachium nipponense*, (Crustacea; Decapoda; Palaemonidae) is an important economic freshwater species in China (Fu et al., 2012). The annual aquaculture production was gradually increased to 205.010 tons in 2016 in China with an output value of over 20 billion. Sexual precocity is the main problem in *M*. *nipponense* aquaculture, restricting the sustainable development of the *M*. *nipponense* industry. Our previous study has been proven that testis was matured at 19 days after metamorphosis (Post-larval developmental stage 19, PL19) (Jin et al., 2016). Testis began to be distinguished at PL13, and sperms can be seen for the first time in the testis at PL19. Thus, inbreeding will be happened between the new born shrimps, leading to the short life span, small size, and low disease resistance. Thus, the long-term goal of *M*. *nipponense* aquaculture is to establish a technique to regulate the testis development in *M. nipponense*. Therefore, a full understanding of the mechanism of male sexual development of *M*. *nipponense* is urgently needed.

The androgenic gland is a special tissue in crustacean species, which plays essential roles in male sexual differentiation and development in crustacean species. The androgenic gland and its secreted hormones have been proven to promote the testes development, male sexual differentiation, and the male sexual characteristics development in crustacean species (Sagi et al., 1986, 1990). The *IAG* gene is an important hormone, secreted by the androgenic gland, playing essential roles in male sexual differentiation and development in crustacean species (Ventura et al., 2009, 2011; Rosen et al., 2010). Knockdown in the expression of *IAG* in *M*. *rosenbergii* resulted in the sex reversal (Ventura et al., 2012). The previous studies also confirmed the multiple and important functions of testis in male sexual differentiation, sexual maturity, and reproductive capability in *M*. *nipponense* (Qiu et al., 1995; Yang et al., 1999; Cao, 2006; Guo, 2007). The histological observation of gonad development during the post-larval development stages of *M*. *nipponense* indicated that the development of the androgenic gland had important regulatory roles in the testis development in *M*. *nipponense* (Jin et al., 2016).

Our previous study predicted that Succinate dehydrogenase complex iron sulfur subunit B (*SDHB*) may play essential roles in male sexual development in *M*. *nipponense* through promoting ATP generation because it was differentially expressed in the testis and the androgenic gland between the reproductive season and non-reproductive season, and was enriched in the metabolic pathways of oxidative phosphorylation and tricarboxylic acid cycle (Jin et al., 2020). Oxidative phosphorylation and tricarboxylic acid cycle were the most enriched metabolic pathways in the transcriptome profiling analysis of the testis and androgenic gland between the reproductive season and non-reproductive season (Jin et al., 2020). *SDHB* is one of four protein subunits that form succinate dehydrogenase (SDH), which catalyzes the oxidation of succinate, playing essential roles in pro-inflammatory response (Kita et al., 1990; Au et al., 1995). The specific structure of iron-sulfur protein was proven to be responsible for the oxidative stress in oxidative phosphorylation. It is also the only enzyme to be involved in the tricarboxylic acid cycle through association with the inner mitochondrial membrane. It is believed that succinate dehydrogenase complex is bound to two small integral membrane proteins of 13.5 and 15.5 kDa through iron protein subunit, which played vital roles in the electron transport chain. Spermatogenic cells of *Lymnaea stagnalis* possessed the succinate dehydrogenase, which prevent the metabolic acidosis in the testis resulted from the anaerobic production of lactate and succinate by Sertoli cells. It also played essential roles in developing rat testis (Gaździk et al., 1986). *SDHB* also had multiple functions, including the resistance to carboxin in Ustilago maydis (Broomfield and Hargreaves, 1992), the prevention of superoxide generation, and premature aging in *C. elegans* (Huang and Lemire, 2009), resistance to boscalid in *Sclerotinia sclerotiorum* (Wang et al., 2015).

In this study, we aimed to verify the important role of *SDHB* in male sexual development mechanism in *M*. *nipponense*, by using rapid amplification of cDNA ends (RACE) cloning, qPCR analysis, western-blot and *in situ* hybridization analysis. This study analyzes the male sexual development from energy metabolism for the first time, providing new insight for the analysis of male sexual development in *M*. *nipponense* and the whole crustacean species.

## Materials and Methods

### Ethics Statement

The Institutional Animal Care and Use Ethics Committee of the Freshwater Fisheries Research Center, Chinese Academy of Fishery Sciences (Wuxi, China) was used to approve all experiments involving *M*. *nipponense* in this study.

### Sample Preparation

A total of 100 healthy adult *M*. *nipponense* were obtained from Tai Lake in Wuxi, China (120°13’44“E, 31°28’22”N), maintained in the lab condition for at least 72 h before tissue collection with the dissolved oxygen of 6 mg/L. Different tissues for qPCR analysis included hepatopancreas, testis, muscle, ovary, gill, eyestalk, heart, and brain. Specimens for the qPCR analysis of different developmental stages were collected from the full-sibs population, collected with their maturation process. The characteristics of various phases of the ovarian reproductive cycle have been well described in the previous report (Qiao et al., 2015). The reproductive season of testis and the androgenic gland were collected in July in summer for western-blot analysis with the water temperature of ≥28°C and illumination time of ≥15 h, while the non-reproductive season of tests and androgenic gland were collected in January in winter with the water temperature of ≤15°C and illumination time of ≤10 h. The samples were washed by phosphate buffer saline (PBS), and immediately frozen in liquid nitrogen until being used for RNA and protein extraction to prevent total RNA and protein degradation.

### Rapid Amplification of cDNA Ends (RACE)

The procedures for RACE cloning have been well-described in previous studies (Jin et al., 2014, 2018). Briefly, RNAiso Plus Reagent (Takara Bio Inc.) was used to extract the total RNA from testis, and then the extracted total RNA was used to synthesize the templates for 3′cDNA and 5′cDNA cloning by using 3′-Full RACE Core Set Ver.2.0 kit and the 5′-Full RACE kit (Takara Bio Inc., Japan). Primer-BLAST tool in NCBI^1^ was used to design the specific primers used for *Mn-SDHB* cloning (Table 1). The BLASTX and BLASTN search program^2^ and the ORF Finder tool^3^ were employed to analyze the structural characteristics. The phylogenetic tree was constructed by MEGA X, followed by the maximum-likelihood method with Bootstrap method of 1000 replications. The species used for the construction of phylogenetic tree were listed in Table 2.

**TABLE 1:** Universal and specific primers used in this study.

**Primer name**	**Nucleotide sequence(5′→3′)**	**Purpose**
*SDHB*-3GSP1	AAGTACCTGGGACCCGCTGT	FWD first primer for *SDHB* 3′ RACE
*SDHB*-3GSP2	AAAGACTGCGATCCTTTCTC	FWD second primer for *SDHB* 3′ RACE
*SDHB* -5GSP1	TCCCTGCTTCTCGGGATCCCA	RVS first primer for *SDHB* 5′ RACE
*SDHB* -5GSP2	TCCACTTGCTCTGCTGCTGCA	RVS second primer for *SDHB* 5′ RACE
3′RACE OUT	TACCGTCGTTCCACTAGTGATTT	RVS first primer for 3′ RACE
3′RACE IN	CGCGGATCCTCCACTAGTGATTTCACTATAGG	RVS second primer for 3′ RACE
5′RACE OUT	CATGGCTACATGCTGACAGCCTA	FWD first primer for 5′ RACE
5′RACE IN	CGCGGATCCACAGCCTACTGATGATCAGTCGATG	FWD second primer for 5′ RACE
*SDHB* -RTF	ACCGCAAGAAGTTGGATGGT	FWD primer for *SDHB* expression
*SDHB* -RTR	TCGATGATCCAACGGTAGGC	RVS primer for *SDHB* expression
EIF-F	CATGGATGTACCTGTGGTGAAAC	FWD primer for EIF expression
EIF-R	CTGTCAGCAGAAGGTCCTCATTA	RVS primer for EIF expression
IAG-RTF	CGCCTCCGTCTGCCTGAGATAC	FWD primer for IAG expression
IAG-RTR	CCTCCTCCTCCACCTTCAATGC	RVS primer for IAG expression
*SDHB* anti-sense Probe	GGAGCGTATAGCAATCAGACGGCAGGCACTG	Probe for *SDHB* ISH analysis
*SDHB* sense Probe	CAGTGCCTGCCGTCTGATTGCTATACGCTCC	Probe for *SDHB* ISH analysis
*SDHB* RNAi-F	TAATACGACTCACTATAGGGACCAGCAGAGCAAGTGGAAT	FWD primer for RNAi analysis
*SDHB* RNAi-R	TAATACGACTCACTATAGGGCCAAGGCTGGATTGATCTGT	RVS primer for RNAi analysis

**TABLE 2:** Species used for phylogenetic tree analysis.

**Species**	**Accession number**
*Macrobrachium nipponense*	MW366891
*Penaeus vannamei*	XP_027220175.1
*Procambarus clarkia*	AVN99065.1
*Hyalella Azteca*	XP_018011988.1
*Armadillidium nasatum*	KAB7496390.1
*Trinorchestia longiramus*	KAF2369055.1
*Orchesella cincta*	ODN01472.1
*Cryptotermes secundus*	XP_023715817.1
*Athalia rosae*	XP_012262627.1
*Kryptolebias marmoratus*	XP_017266916.1
*Anoplopoma fimbria*	ACQ58465.1
*Oreochromis aureus*	XP_031584138.1

### qPCR Analysis

The mRNA expressions of *Mn-SDHB* were measured by qPCR analysis. The Bio-Rad iCycler iQ5 Real-Time PCR System (Bio-Rad) was used to carry out the SYBR Green RT-qPCR assay. The previous studies have well described the detailed procedures of qPCR analysis (Jin et al., 2014, 2018). The primers used for qPCR analysis were listed in Table 1. *EIF* was used as a reference gene in this study (Hu et al., 2018). The amplification efficiency between the *Mn-SDHB* and *EIF* were measured, and the amplification efficiency is the same in this study. The relative mRNA expressions of *Mn-SDHB* were measured by using 2^–ΔΔ*CT*^ method.

### *In situ* Hybridization

The mRNA locations of *Mn-SDHB* were analyzed by using *in situ* hybridization. The different tissues included various reproductive cycles of the ovary, the testis, and androgenic gland in reproductive season. The anti-sense and sense probes of CISH (Chromogenic *in situ* hybridization) study with DIG signal were designed by using Primer5 software based on the cDNA sequence of *Mn-SDHB* (Table 1). Shanghai Sangon Biotech Company synthesized the probes. The previous studies have well described the detailed procedures of *in situ* hybridization (Jin et al., 2018). Slides were examined under light microscope for evaluation.

### Western-Blot Analysis

Testis and androgenic gland samples (20 mg) were respectively obtained in the reproductive season and non-reproductive season. The Bradford method was used to quantify the total protein concentration (Bradford, 1976). The detailed procedure of western-blot was well described in a previous study (Sun et al., 2017). Briefly, a 10% SDS-polyacrylamide (SDS-PAGE) was used to separate 50 mg protein from each sample, and then transferred to a PVDF membrane (Millipore, Bedford, MA, United States).

### RNA Interference (RNAi) Analysis

The potentially regulatory roles of *Mn-SDHB* on male sexual development in *M*. *nipponense* were analyzed by using RNAi. Snap Dragon tools,^4^ which were used to design the specific RNAi primer with T7 promoter site, shown in Table 2. The Transcript Aid^TM^ T7 High Yield Transcription kit (Fermentas, Inc, United States) was used to synthesize the *Mn-SDHB* dsRNA, following the procedures of the manufacturer. A total of 300 healthy mature male *M*. *nipponense* were collected with body weights of 3.12–4.87 g, and divided into two groups. As described in previous study (Jiang et al., 2014; Li et al., 2018), the prawns from experimental group were injected with 4 μg/g *Mn-SDHB* dsRNA. The concentration of the *Mn-SDHB* dsRNA was adjusted to 4 μg/μl. Thus, the injected volume of *Mn-SDHB* dsRNA into each prawn was the same as that of their body weight, while the prawns from the control group were injected with equal volume of vehicles based on the body weight. The *SDHB* mRNA expression was investigated in the testis by qPCR after the injection of 1, 7, and 14 days, in order to detect the interference efficiency (*N* ≥ 5).

### Histological Observation

The morphological changes of the testis between different days after RNAi treatment were observed by Hematoxylin and eosin (HE) staining. Five testicular samples were respectively collected after 1, 4, 7, 10, and 14 days of RNAi treatment for HE staining. The procedures have been well described in previous studies (ShangGuan et al., 1991; Ma et al., 2006). Olympus SZX16 microscope was used to observe the slides (Olympus Corporation, Tokyo, Japan). The various cell types were labeled following the description of the previous report (Jin et al., 2016).

### Statistical Analysis

SPSS Statistics 23.0 was used to measure the statistical differences, estimated by one-way ANOVA followed by LSD and Duncan’s multiple range test. *p* < 0.05 was used to indicate statistical significance. Quantitative data were expressed as mean ± SD.

## Results

### Genome and cDNA Sequence Analysis

**FIGURE 1:**
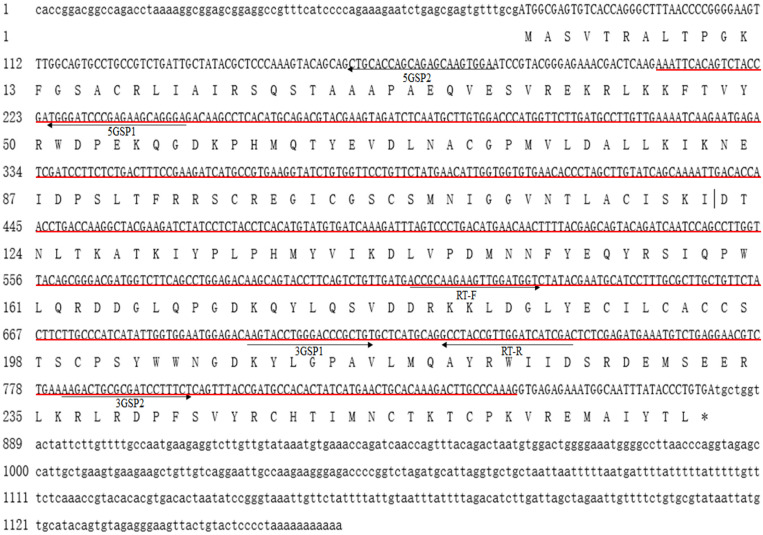
Nucleotide and deduced amino acid sequence of *Mn-SDHB*. The nucleotide sequence is displayed in the 5′–3′ directions and numbered at the left. The deduced amino acid sequence is shown in a single capital letter amino acid code. 3′ UTR and 5′ UTR are shown with lowercase letters. Codons are numbered at the left with the methionine (ATG) initiation codon, an asterisk denotes the termination codon (TGA). Red line indicated the PLN00129 superfamily.

**FIGURE 2:**
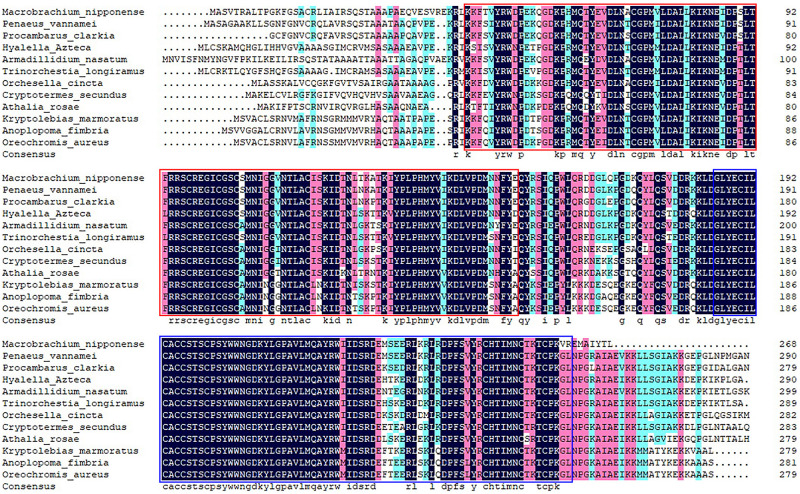
The similarity identity of amino acid sequences of *SDHB* between different species. Red box indicates the 2Fe–2S iron–sulfur cluster binding domain. Blue box indicates the 4Fe–4S dicluster domain.

The full-length cDNA sequence of *Mn-SDHB* was 1,268 base pairs (bp) long with an open reading frame of 807 bp, encoding for 268 amino acids. The 5′ untranslated region (UTRs) and the 3′ UTR were 74 bp and 387 bp long, respectively. The sequence has been submitted to NCBI with the accession number of MW366891. The molecular weight and theoretical isoelectric point of the protein were 30.684 kDa and 8.68, respectively. The full-genome sequence of *Mn-SDHB* was 54,608 bp at Chromosome 34 at position 47,658,691-47,713,299, including 7 introns and 6 exons. One PLN00129 superfamily was detected in the cDNA sequence of *Mn-SDHB* (Figure 1). In addition, *Mn-SDHB* contained a 2Fe–2S iron–sulfur cluster binding domain (44–152 aa) and a 4Fe–4S dicluster domain (185–261 aa) (Figure 2).

**FIGURE 3:**
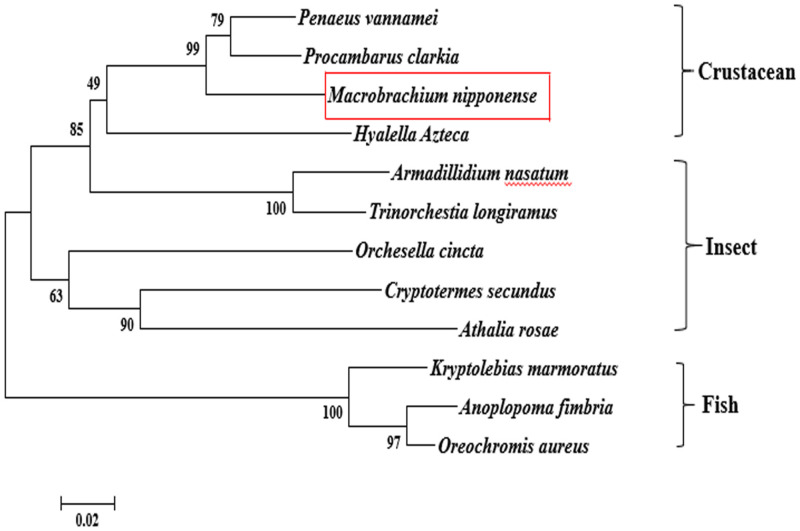
The phylogenetic tree of *SDHB* from different organisms based on amino acid sequence comparisons. Species names of *SDHB* are listed on the right of the tree. Red rectangles indicated *M. nipponense*.

According to the BLASTN similarity in NCBI, the similarities of *Mn-SDHB* and *SDHB* in other species were over 70%, including *Salmo salar* (74.74%), *Salvelinus alpinus* (74.55%), *Oncorhynchus kisutch* (74.20%), and *Megachile rotundata* (73.99%) (Figure 2). MEGA 5.1 was further used to analyze the evolutionary relationship between the amino acid sequences of *Mn-SDHB* and other well-defined *SDHB* sequences in NCBI, followed the maximum-likelihood method to construct a condensed phylogenetic tree. According to the phylogenetics tree analysis, the amino acids sequence of *Mn-SDHB* has the closest evolutionary relationship with that of crustacean species, including *Penaeus vannamei* and *Procambarus clarkia*. The amino acids sequence of *Mn-SDHB* was then clustered with the amino acid sequences of insect species as a group, while *Mn-SDHB* has a long evolutionary relationship with that of fish species (Figure 3).

### Expression Analysis in Different Tissues and Developmental Stages

**FIGURE 4:**
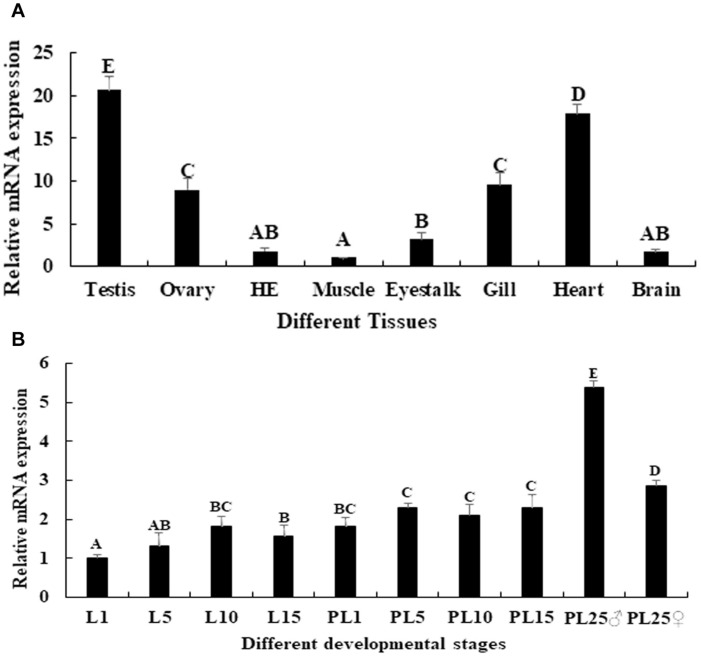
Expression characterization of *Mn-SDHB* in different mature tissues and developmental stages. The amount of *Mn-SDHB* mRNA was normalized to the *EIF* transcript level. Data are shown as mean + SD (standard deviation) of tissues from three separate individuals. Capital letters indicate expression difference between different samples. **(A)** Expression characterization in different mature tissues. **(B)** Expression characterization in different developmental stages. HE, hepatopancreas.

The physiological function of a gene may be reflected by the mRNA tissue distribution, evaluated by qPCR analysis. The qPCR analysis of *Mn-SDHB* in different tissues revealed that the highest RNA expression of *Mn-SDHB* was observed in the testis, and showed significant difference with other tested tissues (*p* < 0.05), followed by the heart, gill and ovary. The lowest mRNA expression of *Mn-SDHB* was observed in muscle. The expression in testis was 20.66-folder and 2.3-folder higher than that of muscle and ovary, respectively (Figure 4A).

qPCR was further used to evaluate the mRNA expression of *Mn-SDHB* in different larval and post-larval developmental stages. The mRNA expression of *Mn-SDHB* was gradually increased from larval developmental stage day 1 (L1) to L10, and reached the peak at L10 during the larval developmental stages. The expression at L10 was 1.83-fold higher than that of L1, and showed significant difference (*p* < 0.05) (Figure 4B). The expression of *Mn-SDHB* was significantly increased during the post-larval developmental stages, compared with that of larval developmental stages (*p* < 0.05). The expression remained stable from post-larval developmental stage 1 (PL1) to PL15, and showed no significant difference (*p* > 0.05). The new born prawns can be distinguished between male and female for the first time in PL25. The expressions were dramatically increased in both PL25♂ (male) and PL25♀ (female), and showed significant difference with other tested developmental stages. However, the expression in PL25♂ was almost 2-fold higher than that of PL25♀ (Figure 4B).

### Western-Blot Analysis

**FIGURE 5:**
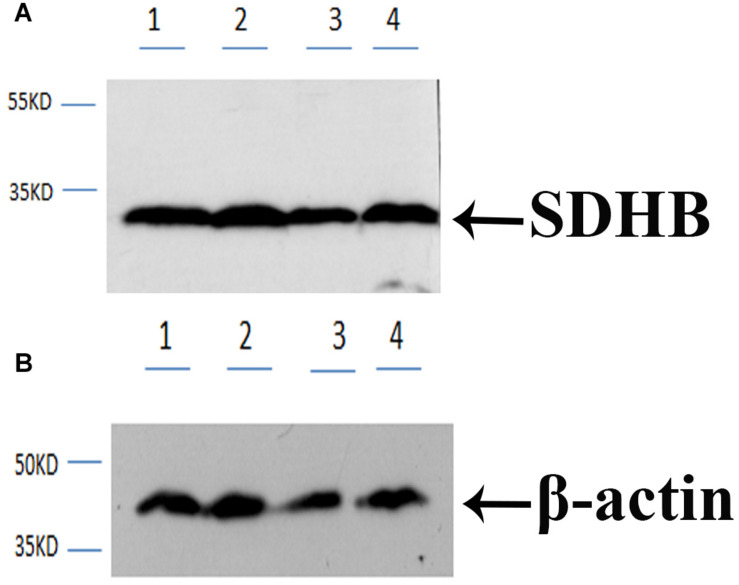
Western-blot analysis in testis and androgenic gland from reproductive season and non-reproductive season. **(A)** Western-blot for SDHB. **(B)** Western-blot for B -action. Line 1: Testis from the non-reproductive season; Line 2: Testis from the reproductive season; Line 3: Androgenic gland from the non-reproductive season; Line 4: Androgenic gland from the reproductive season. B -action was used as reference gene.

Western-blot analysis was performed in the testis and androgenic gland of reproductive season and non-reproductive season. The molecular mass of *Mn-SDHB* was approximately 30 kDa, according to the western-blot analysis, which was similar to the predicted molecular weight by software. The previous study has indicated that the mRNA expressions of *Mn-SDHB* in the testis and androgenic gland were higher than that of non-reproductive season (Jin et al., 2020). As shown in Figure 5, clear bands were observed at approximately 30 kDa in the testis and androgenic gland from both of the reproductive season and non-reproductive season, indicating the *Mn-SDHB* protein was transcribed in these tissues. It is observed that the protein expression levels of *Mn-SDHB* were up-regulated in the testis and androgenic gland from reproductive season than that in non-reproductive season, which were consistent with that of qPCR analysis. Clear bands were also observed in control group, using β-actin as a reference gene.

### Expression Analysis in Different Reproductive Cycle of Ovarian Development

**FIGURE 6:**
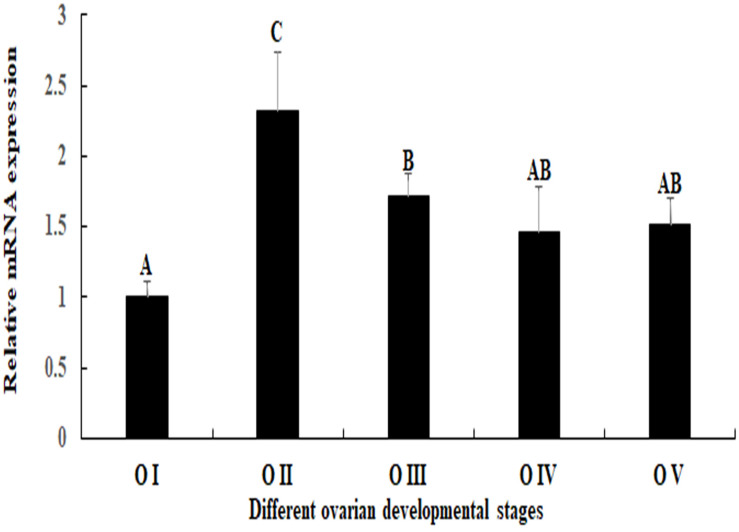
Expression characterization of *Mn-SDHB* in different reproductive cycles ovary. The amount of *Mn-SDHB* mRNA was normalized to the *EIF* transcript level. Data are shown as mean + SD (standard deviation) of tissues from three separate individuals. Capital letters indicate expression difference between different samples.

*Mn-SDHB* was expressed during the whole ovarian reproductive cycle. The lowest expression level of *Mn-SDHB* was observed in O I, while the expression of *Mn-SDHB* reached the peak at O II. The expression in O II is more than 2-folder higher than that of O I, which showed significant difference with other ovarian developmental stage (*p* < 0.05). The expression level of *Mn-SDHB* was then gradually decreased from O III to O V, while the expressions showed no significant difference (*p* > 0.05) (Figure 6).

### *In situ* Hybridization Observation

**FIGURE 7:**
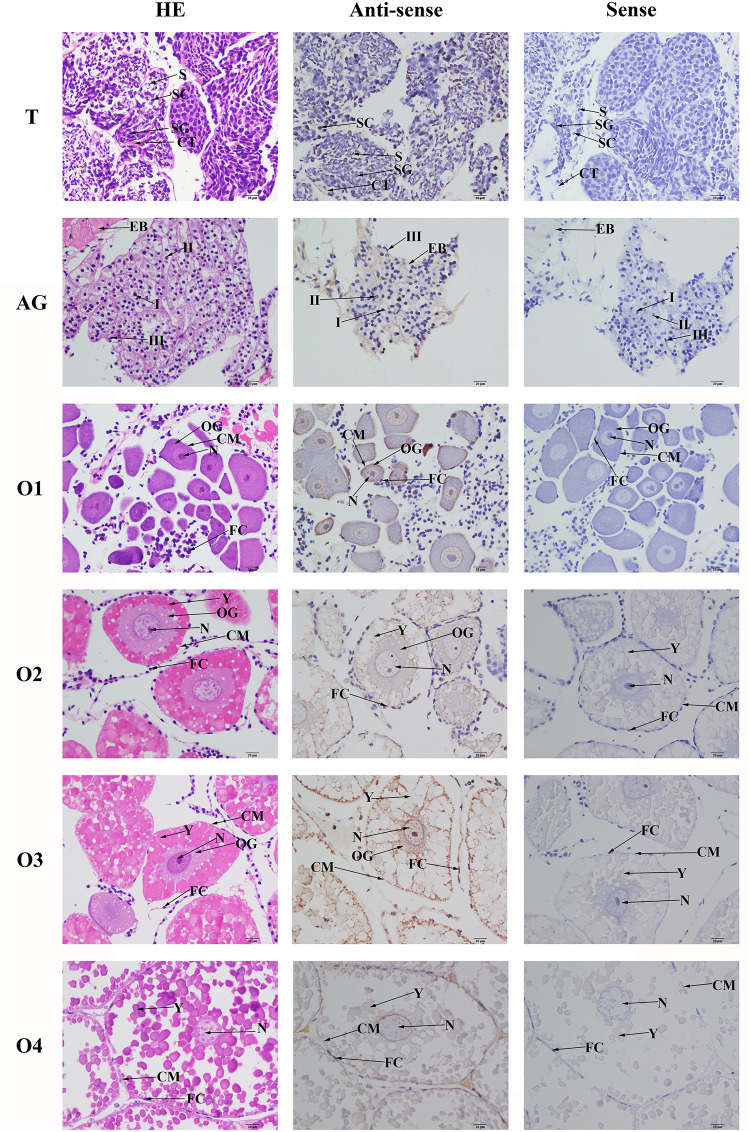
*In situ* hybridization analysis of *Mn-SDHB* gene in testis and androgenic gland from reproductive season, and different ovarian reproductive cycle of *M*. *nipponense*. ST, seminiferous tubule; SG, Spermatogonia; SC, spermatocyte; S, sperm; CT, collecting tissue; E, wall epithelium; EM, eosinophilic matrix; VD, vas deferens; EB, ejaculatory bulb; OG, oogonium; OC, oocyte; CM, cytoplasmic membrane; N, nucleus; Y, yolk granule; FC, follicle membrane. Scale bars = 20 μm.

*In situ* hybridization analysis was performed in order to detect the locations of *Mn-SDHB* mRNA in the testis, androgenic gland, and different reproductive cycles of ovarian development. Hematoxylin and eosin (HE) staining showed that the cells in the testis included spermatogonia, spermatocyte, sperms, and collecting tissues. mRNA signals of *Mn-SDHB* were observed in spermatogonium (Figure 7). Androgenic gland consisted of androgenic gland cells and the ejaculatory bulb. Strong mRNA signals *Mn-SDHB* in androgenic gland were only observed in the ejaculatory bulb, while no signals were found in androgenic gland cells (Figure 7).

Oogonia and follicle cells were observed in ovary stage I, which are derived via differentiation from ovarian epithelial cells, the follicular cavity formed in stage II, which were derived from the follicle cells. Oocyte volume gradually increased in stage III, and yolk granules accumulated in the oocyte (stage IV) during oogenesis and vitellogenesis. According to the Figure 6, the mRNA signals of *Mn-SDHB* were observed in all of the cell types from O I to O IV, including nucleus, oogoniums, oocytes, cytoplasmic membrane, yolk granule, follicle cells, and follicle membrane (Figure 7).

### RNAi Analysis

**FIGURE 8:**
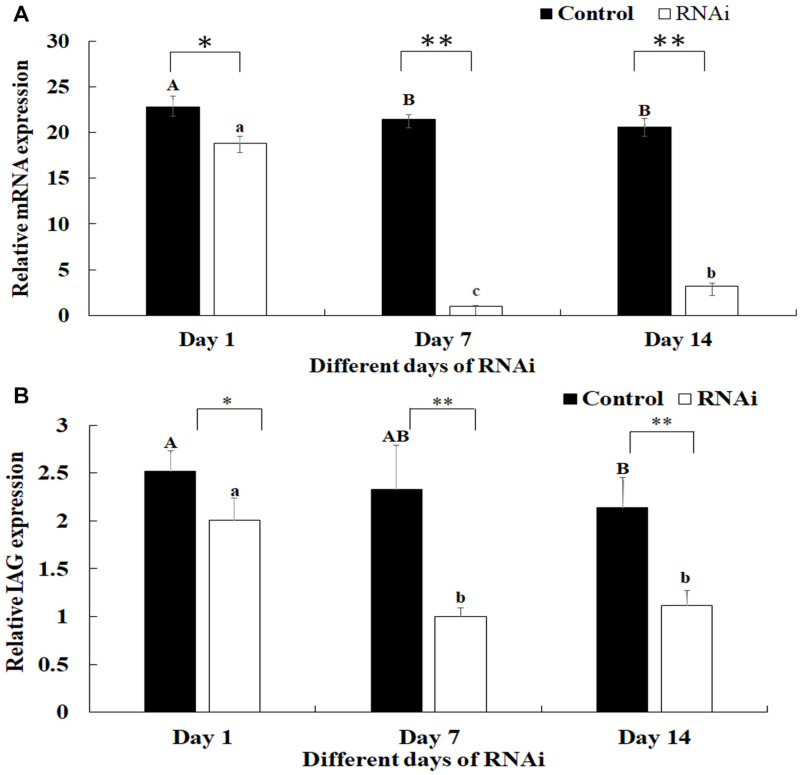
Expression characterization of *Mn-SDHB* and *Mn-IAG* at different days after *Mn-SDHB* dsRNA injection. The amount of *Mn-SDHB and Mn-IAG* mRNA was normalized to the *EIF* transcript level. Data are shown as mean + SD (standard deviation) of tissues from three separate individuals. Capital letters indicate expression difference between different days after vehicle injection in control group. Lowercase indicate expression difference between different days after *Mn-SDHB* dsRNA injection in RNAi group. * (*P* < 0.05) and ** (*P* < 0.01) indicates significant expression difference between the RNAi group and control group at the sample day. **(A)** Expression characterization of *Mn-SDHB* at different days after *Mn-SDHB* dsRNA injection. **(B)** Expression characterization of *Mn-IAG* at different days after *Mn-SDHB* dsRNA injection.

The mediated functions of *Mn-SDHB* in male sexual development was evaluated by using RNAi analysis in male prawns. The *Mn-SDHB* dsRNA was injected twice and the RNAi experiment lasted for 2 weeks. The *Mn-SDHB* mRNA expression remained at a stable level during the 2 weeks in control group. However, the *Mn-SDHB* mRNA expressions in the RNAi group were significantly decreased at day 7 and day 14, compared with that in day 1. The mRNA expressions of *Mn-SDHB* were respectively decreased over 95 and 85% at day 7 and day 14 in the RNAi group, compared to those in the control group (*P* < 0.01) (Figure 8A). The mRNA expression of *Mn-IAG* was also measured in the androgenic gland from the same prawn, treated by *Mn-SDHB* dsRNA. The expressions of *Mn-IAG* were decreased with the decrease of *Mn-SDHB*. The expressions were respectively decreased by about 57 and 48% at day 7 and day 14 in the RNAi group, compared with those in the control group at the same day (*P* < 0.01) (Figure 8B).

**FIGURE 9:**
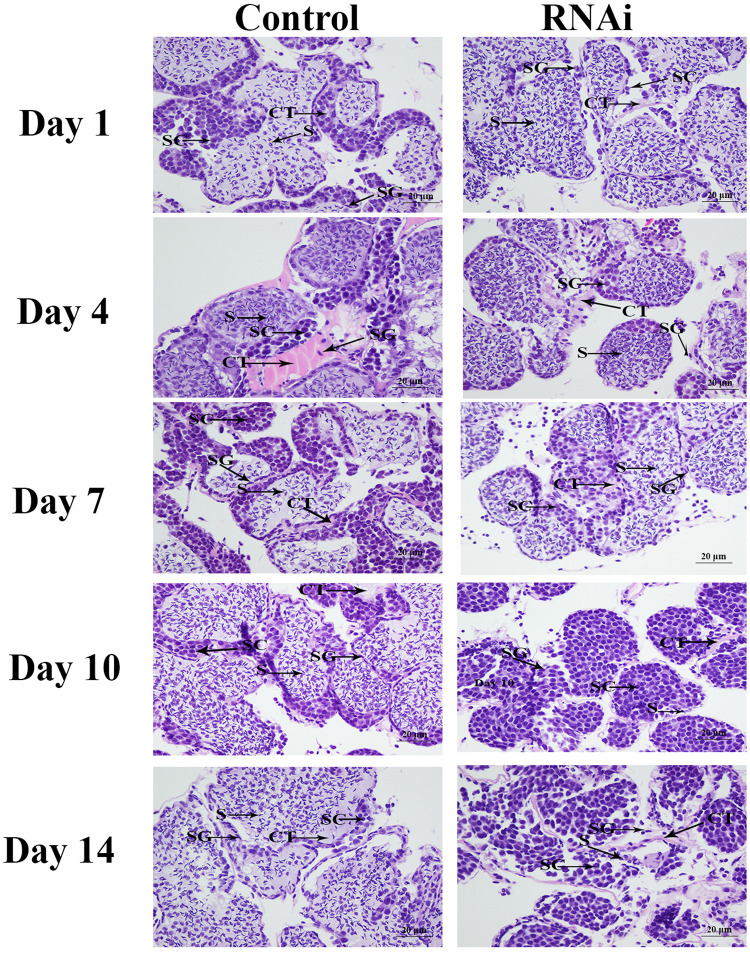
The histological observations of testis between RNAi and control group. SG, Spermatogonia; SC, spermatocyte; S, sperm; CT, collecting tissue. Scale bars = 20 μm.

The histological observations of testis revealed that the majority of cells in the testis from control group were sperms, while only a few of spermatogonias and spermatocytes were observed. In RNAi group, the number of sperms were deceased with the time of *Mn-SDHB* dsRNA treatment, while the number of spermatogonias were increased. Sperms were rarely found at day 14 after *Mn-SDHB* dsRNA treatment (Figure 9).

## Discussion

The previous studies have been proven that SDH plays vital roles in the immune system and immune response to infection (Mills et al., 2016; Pinegin et al., 2018). *SDHB* is a component of SDH, and has been proven to be an important gene in the ATP-releasing process. It also proved to have a function in testis development of *Lymnaea stagnalis* and rat (Gaździk et al., 1986). *SDHB* was predicted to be involved in the male sexual differentiation and development in *M*. *nipponense* (Jin et al., 2020). In this study, we aimed to focus on the potential functions of *SDHB* in male sexual differentiation and development through performing the qPCR, *in situ* hybridization and RNAi analysis in male prawns, combined with the histological observations.

BLASTN similarity comparisons revealed over 70% between *Mn-SDHB* and *SDHB* in other crustacean, insect, and fish species, indicating *SDHB* is a conserved gene. In addition, *Mn-SDHB* also contained a 2Fe–2S iron–sulfur cluster binding domain and a 4Fe–4S dicluster domain, which was consistent with a previous study (Sun et al., 2020). *Mn-SDHB* has the closest evolutionary relationship with amino acid sequences of *SDHB* from crustacean species, based on the maximum-likelihood analysis. These crustacean species include *Penaeus vannamei* and *Procambarus clarkia*. *Mn-SDHB* was then clustered with *SDHB* from insect species as a group, while the evolutionary relationship of *Mn-SDHB* with that of fish species was dramatically long. This is consistent with that of taxonomy.

qPCR analysis was used to detect the expression level of *Mn-SDHB* in various tissues and developmental stages. The previous studies showed that *SDH* performs multiple biological functions and exists in diverse vertebrate tissues and species (Hochachka et al., 1975; Hochachka and Dressendorfer, 1976; Chouchani et al., 2014). qPCR analysis was performed in *Apostichopus japonicus*, and the different tissues included coelomocytes, muscle, tentacle, respirating trees, and intestine. The highest expression level was observed in muscle, implying its high mitochondrial content and high oxidative potential in muscles (Sun et al., 2020). In this study, *Mn-SDHB* was detected in all tested tissues, while the highest expression levels were detected in the testis, and the expression level in the ovary was relatively high. However, the expression in the testis was 2.3-folder higher than that of the ovary. These results implied that the gonad may have high mitochondrial content and high oxidative potential, especially for that in the testis. The qPCR analysis in different developmental stages revealed that the mRNA expression levels of *Mn-SDHB* in post-larval developmental stages were generally higher than that of larval-developmental stages. The post-larval developmental stages have been proven to be the sensitive period for the gonad differentiation and development in *M*. *nipponense* (Jin et al., 2016). The new-born male and female prawn can be distinguished for the first-time at PL25 by morphological observation. The *Mn-SDHB* expressions were significantly increased at both of PL25♂ and PL25♀, while the expression in PL25♂ was almost 2-folder higher than that of PL25♀. Thus, *SDHB* was considered to have important roles in gonad development in *M*. *nipponense*, especially in the testis through combination of the qPCR results of different mature tissues and different developmental stages. According to the western-blot analysis, clear bands can be observed in the testis and androgenic gland from both the reproductive season and non-reproductive season, indicating that the *Mn-SDHB* protein can be transcribed in these tissues. The bands of testis and androgenic gland from the reproductive season were much clearer than those of the non-reproductive season, indicating that the protein expressions of *Mn-SDHB* were up-regulated in the reproductive season, compared with those of the non-reproductive season, which is consistent with those of mRNA expression (Jin et al., 2020).

The expression levels of *Mn-SDHB* were also detected during the different reproductive cycle of ovarian development. The *Mn-SDHB* mRNA expressions reached the peak at O II, and then gradually decreased from O III to O V, and the lowest expression level was observed in O I. According to the histological observation, the follicular cavities, derived from the follicle cells, were formed in stage II (Li et al., 2018). A reasonable explanation for the highest expression in O II was that *Mn-SDHB* plays essential roles in activating the ovarian development, especially for the formation of the follicular cavity.

*In situ* hybridization was also used to detect the mRNA locations of *Mn-SDHB* in the testis, androgenic gland, and different reproductive cycles of ovarian development. To the best of our knowledge, the *in situ* hybridization analysis of *SDHB* was not reported in any species. According to the *in situ* hybridization analysis, strong signals were observed in the spermatogonia and collecting tissues of testis, while no signals were observed in sperms, suggesting that *Mn-SDHB* plays essential roles in promoting the activation of testis development (Jin et al., 2018). No *Mn-SDHB* mRNA signals were observed in the androgenic gland cell, indicating that the androgenic gland did not directly secrete the *SDHB*. However, the strong signals in the ejaculatory bulb surrounding the androgenic gland cells indicated that *SDHB* plays essential roles in maintaining the normal structure and function of the androgenic gland, promoting and supporting the formation of androgenic gland cells (Jin et al., 2018). Strong mRNA signals of *Mn-SDHB* were observed in all cell types of the ovary, implying that *SDHB* was involved in the whole reproductive cycle of ovarian development.

RNAi can inhibit the gene expression or translation through the short double-stranded (ds) RNA molecules in the cell’s cytoplasm (Kusaba, 2004; Jones, 2005; Smith et al., 2009). RNAi has been widely used in gene function analysis in *M*. *nipponense* (Li et al., 2015, 2018; Jin et al., 2018). Previous studies have proven that *SDH* driven-mROS plays an important role in the development of antimicrobial innate immune responses (Pinegin et al., 2018), enhancing the host defense during infection (Mills et al., 2016). siRNA was used to knockdown the *SDHB* expression in *A. japonicus*, leading to the decrease of the mitochondrial membrane potential and further the decrease of mitochondrial ROS production in *A. japonicus* coelomocytes (Sun et al., 2020). However, a previous study predicted that *SDHB* may be involved in the male sexual development in *M*. *nipponense* (Jin et al., 2020). RNAi was used to analyze the potential functions of *SDHB* in male sexual development in *M*. *nipponense*. The mRNA expressions of *Mn-SDHB* were significantly decreased at day 7 and day 14 after the treatment of *Mn-SDHB* dsRNA, indicating that the Mn-SDHB dsRNA in this study was efficient to knockdown the *Mn-SDHB* expression. The expression levels of *Mn-IAG* were also measured in the androgenic gland from the same prawn, treated by *Mn-SDHB* dsRNA. The expressions of *Mn-IAG* were decreased with the decrease of *Mn-SDHB*, indicating that *SDHB* has a positive regulatory relationship with that of *IAG* in *M*. *nipponense*. The *IAG* gene has been proven to play essential roles in male sexual differentiation and development in crustacean species (Ventura et al., 2009, 2011, 2012; Rosen et al., 2010). The positive regulatory relationship between *SDHB* and *IAG* indicated that *SDHB* was involved in the male sexual development in *M*. *nipponense*. The morphological differences of testis between the RNAi group and control group were measured by histological observations. According to the morphological difference analysis of testis between the RNAi and control group, the sperms were decreased with the time of *Mn-SDHB* dsRNA treatment, and sperms were hardly observed at day 14 of *Mn-SDHB* dsRNA treatment, while spermatogonias were dramatically increased, indicating that *Mn-SDHB* has positive effects on testis development in *M*. *nipponense*.

In conclusion, we further analyzed the functions of *SDHB in M. nipponense* in this study, based on the previous study. Our data strongly suggest that *Mn-SDHB* played essential roles in gonad differentiation and development, especially for that in male sexual development. *Mn-SDHB* was proven to have positive regulatory effects on the testis development, based on the *Mn-SDHB* dsRNA treatment, combined with the histological observations. This study analyzes the male sexual development from energy metabolism for the first time in *M*. *nipponense*, promoting the future studies of male sexual development in other crustacean species as well.

## Data Availability Statement

The datasets presented in this study can be found in online repositories. The names of the repository/repositories and accession number(s) can be found in the article/supplementary material.

## Ethics Statement

The animal study was reviewed and approved by Macrobrachium nipponense Freshwater Fisheries Research Center. Written informed consent was obtained from the owners for the participation of their animals in this study.

## Author Contributions

ShJ designed the experiment. YH performed the RNAi analysis. HF supervised the experiment. SuJ provided the experimental prawns. YX performed the western-blot analysis. HQ performed the qPCR analysis. WZ performed the *in situ* hybridization analysis. YG performed the histological observations. YW cloned the full cDNA sequence. All authors contributed to the article and approved the submitted version.

## Conflict of Interest

The authors declare that the research was conducted in the absence of any commercial or financial relationships that could be construed as a potential conflict of interest.

## Footnotes

1 http://www.ncbi.nlm.nih.gov/tools/primer-blast/

2 http://www.ncbi.nlm.nih.gov/BLAST/

3 http://www.ncbi.nlm.nih.gov/gorf/gorf.html

4 http://www.flyrnai.org/cgibin/RNAifind_primers.pl
